# Capsaicin Ameliorates High-Fat Diet-Induced Atherosclerosis in ApoE^−/−^ Mice via Remodeling Gut Microbiota

**DOI:** 10.3390/nu14204334

**Published:** 2022-10-17

**Authors:** Zijian Dai, Siqi Li, Yantong Meng, Qingyu Zhao, Yiyun Zhang, Zhuoma Suonan, Yuge Sun, Qun Shen, Xiaojun Liao, Yong Xue

**Affiliations:** 1 National Engineering and Technology Research Center for Fruits and Vegetables, College of Food Science and Nutritional Engineering, China Agricultural University, Beijing 100083, China; 2 National Center of Technology Innovation (Deep Processing of Highland Barley) in Food Industry, China Agricultural University, No. 17 Qinghua East Road, Haidian District, Beijing 100083, China

**Keywords:** capsaicin, atherosclerosis, metabolites, gut microbiota, inflammation, single molecule real-time sequencing technology

## Abstract

Capsaicin is a pungent alkaloid abundantly present in peppers with outstanding biological activities, including the anti-atherosclerosis effect. Previous studies revealed that gut microbiota played an important role in the beneficial effects of capsaicin, but whether it is essential for the anti-atherosclerosis effect of capsaicin is unclear. This study evaluated the anti-atherosclerosis effect of capsaicin in ApoE^−/−^ mice and further explored the role of depleting gut microbiota in the improvement of atherosclerosis. The results showed that capsaicin administration could prevent the development of atherosclerosis and improve serum lipids and inflammation, while antibiotic intervention abolished the alleviation of atherosclerosis by capsaicin. In addition, capsaicin administration could significantly increase the abundance of *Turicibacter*, *Odoribacter*, and *Ileibacterium* in feces, and decrease the abundance of deoxycholic acid, cholic acid, hypoxanthine, and stercobilin in cecal content. Our study provides evidence that gut microbiota plays a critical role in the anti-atherosclerosis effect of capsaicin.

## 1. Introduction

Cardiovascular diseases are the leading cause of death globally, and an estimated 17.9 million people died from cardiovascular diseases in 2019, accounting for 32% of all deaths [1]. Atherosclerosis is the main pathological basis for the occurrence of most cardiovascular diseases, which seriously threatens human health and life [2]. According to the World Health Organization, 61% of cardiovascular deaths can be explained by eight risk factors including low fruit and vegetable intake [3]. There is increasing evidence that a considerable amount of bioactive compounds in fruits and vegetables can effectively reduce the formation of atherosclerotic plaques, prevent atherosclerosis-related complications, and thus reduce the risk of cardiovascular diseases [4].

Capsaicin, a pungent alkaloid mainly found in peppers, has a variety of biological activities including anti-obese, anti-inflammatory, glucose-lowering, and lipid-lowering effects [5,6,7,8]. Numerous studies suggested that capsaicin could prevent the development of atherosclerosis mainly due to the improvement of lipid metabolism, inflammation, and endothelial dysfunction [9,10,11,12]. The gut microbial genome, known as the second genome of the host, significantly affects host energy metabolism, regulates intestinal immune responses, and mediates host oxidative stress and inflammatory responses [13]. Recently, it was found that gut microbiota also played a key role in the beneficial effects of capsaicin [14,15]. A previous study showed that the diversity of gut microbiota changed in ob/ob mice after capsaicin administration, and the *Firmicutes*/*Bacteroidetes* ratio and the abundance of genus *Roseburia* (butyrate-producing bacterium) were increased [16]. The correlation analysis showed that the abundance of genus *Roseburia* was negatively correlated with fasting blood glucose and glucose tolerance, suggesting that gut microbiota might contribute to the glucose-lowering effect of capsaicin. Another study reported that capsaicin administration in mice fed with a high-fat diet (HFD) increased the abundance of butyrate-producing bacteria and decreased the abundance of lipopolysaccharide-producing bacteria, and fecal microbiota transplantation experiments further confirmed that capsaicin-induced alteration in gut microbiota was a critical factor for the anti-obesity effect of capsaicin [14]. Although in vitro and in vivo experiments have provided some support for the anti-atherosclerosis effect of capsaicin [9,11,12], it is unclear whether the role of gut microbiota is critical in the improvement of atherosclerosis, and the potential mechanism needs to be further elucidated.

Therefore, our study aimed to investigate the role of gut microbiota in ameliorating the effect of capsaicin on atherosclerosis. We used ApoE knockout (ApoE^−/−^) mice fed a HFD or a HFD supplemented with capsaicin to explore the effects of capsaicin on blood lipids, inflammation, and aortic pathology, and further compared them to the antibiotic-treated mice with capsaicin administration to evaluate whether capsaicin can improve atherosclerosis or not when the gut microbiota is depleted. In addition, gut microbiota composition and cecal metabolites were measured by single molecule real-time (SMRT) sequencing technology and super high performance liquid chromatography tandem mass spectrometry (UHPLC-MS) respectively, to explore the effects of capsaicin on the gut microbiota and cecal metabolites. This research suggests that the gut microbiota plays a critical role in the anti-atherosclerosis effect of capsaicin, which provides a new theoretical basis for the health benefits of capsaicin.

## 2. Materials and Methods

### 2.1. Materials

Capsaicin (98.3% purity) was purchased from DaXingAnLing Lingonberruy Boreal Biotech Co., Ltd. (Songling, China). Triglyceride (TG), total cholesterol (TC), low-density lipoprotein cholesterol (LDL-c), and high-density lipoprotein cholesterol (HDL-c) assay kits were purchased from Nanjing Jiancheng Institute of Bioengineering (Nanjing, China). Interleukin-1β (IL-1β), interleukin-6 (IL-6), tumor necrosis factor-α (TNF-α), and lipopolysaccharide (LPS) assay kits were purchased from Jiangsu Meimian Industrial Co., Ltd. (Yancheng, China).

### 2.2. Animals and Experiment Design

**Figure 1:**
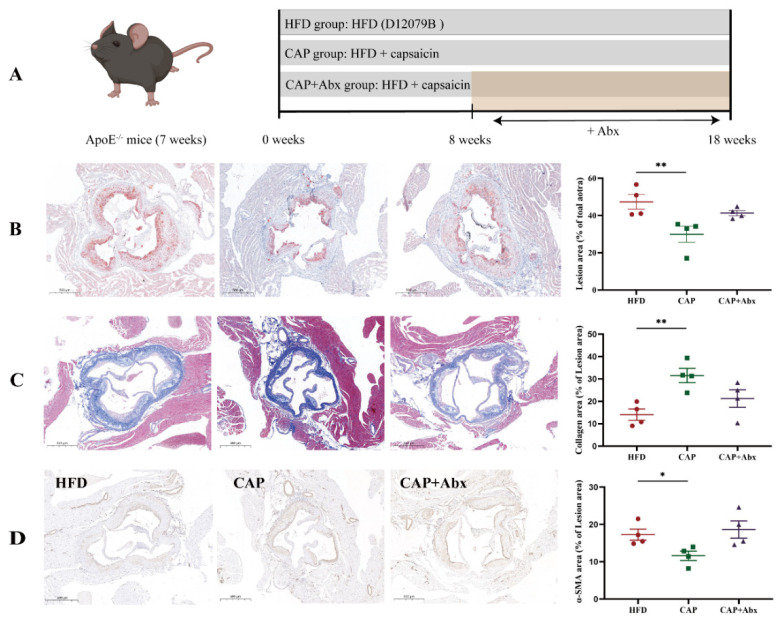
Effects of capsaicin supplementation and antibiotic intervention on the development of atherosclerosis. (**A**) Schema showing the animal groups and treatments. Representative sections and quantitative analyses of plaque area (**B**), collagen (**C**) and α-smooth muscle actin (α-SMA)-positive vascular smooth muscle cells (**D**) in the aortic sinus. Data are expressed as mean ± SEM (*n* = 4). * *p* < 0.05 and ** *p* < 0.01 compared with HFD group. Abx, broad-spectrum antibiotic cocktail; α-SMA, α-smooth muscle actin.

Twenty-four male ApoE^−/−^ mice (7 weeks old) were purchased from Beijing Vital River Laboratory Animal Technology Co., Ltd. (Beijing, China), and were reared in the Specific Pathogen Free (SPF) animal laboratory under controlled temperature (23 ± 2 °C) and relative humidity (55 ± 5%) with a 12 h cycle of light-dark. After one week of acclimatization, the mice were randomly divided into three groups (*n* = 8 per group): (1) HFD group: the mice received a HFD (40 kcal% fat, 0.15% cholesterol, D12079B; Research Diets, New Brunswick, NJ, USA); (2) Capsaicin-treated group (CAP): the mice were fed a HFD containing 0.01% capsaicin (*w*/*w*) (Changzhou Shuyishuer Bio-Tec Co., Ltd., Changzhou, China), the dose of capsaicin was referred to previous studies [9,11]; (3) Antibiotic-treated group (CAP+Abx): the mice were fed a HFD containing 0.01% capsaicin (*w*/*w*) during the intervention period and were fed a broad-spectrum antibiotic cocktail (Macklin Biochemical Co., Ltd., Shanghai, China) containing ampicillin (1 mg/mL), metronidazole (1 mg/mL), neomycin (1 mg/mL) and vancomycin (0.5 mg/mL) in their drinking water during the 8th-18th week of the intervention period. All groups were fed for 18 weeks (Figure 1A). The fecal samples were collected from each mouse in sterile EP tubes in the 17th week, frozen in liquid nitrogen immediately, and stored at −80 °C until further analysis. At the end of the experiment, the mice were anesthetized with pentobarbital sodium and sacrificed. The blood samples were taken from the orbital vascular plexus after 12 h fasting, and the serum was isolated at 4 °C (3500 rpm, 15 min) and then stored at −80 °C until required. The adventitial connective tissue and fat of the whole heart and aorta were gently removed under a dissecting microscope, and the basal portion of the heart and proximal aortic root were excised and harvested. Colon tissue and cecal content were also collected. All the procedures were approved by the Animal Care Committee of China Agricultural University (AW72110202-4) and followed the guidelines of the National Research Council Guidelines.

### 2.3. Biochemical Analysis

The levels of TG, TC, LDL-c, and HDL-c in serum were measured by commercially available kits according to the instructions of the manufacturer (Nanjing Jiancheng Institute of Bioengineering, Nanjing, China).

### 2.4. ELISA Measurement

The levels of IL-1β, IL-6, TNF-α, and LPS in serum were determined by a specific ELISA kit (Jiangsu Meimian Industrial Co., Ltd., Yancheng, China) according to the instructions of the manufacturer.

### 2.5. Atherosclerotic Lesion Analysis

The whole heart was collected and the atherosclerotic lesion size was assessed at the aortic sinus. All of the samples were fixed with 4% paraformaldehyde (*v*/*v*) for more than 24 h. Half of the samples (*n* = 4 per group, samples were randomly selected after the sacrifice) were performed on cryosection for oil red staining. Tissues were placed in a 15% sucrose solution at 4 °C for dehydration, and then transferred to a 30% sucrose solution for dehydration at 4 °C. The dehydrated tissues were slightly dried with filter paper and then embedded in optimal cutting temperature compounds. The sections of 8–10 μm were cut by freezing microtome (CRYOSTAR NX50, Thermo, Waltham, USA), and then dyed with oil red. Finally, the slices were sealed with a glycerol gelatin sealing agent and viewed under a microscope (BX51, Olympus Corporation, Tokyo, Japan). The plaque area was measured using Image-Pro Plus software (Media Cybernetics, Rockville, USA).

The levels of α-smooth muscle actin (α-SMA)-positive vascular smooth muscle cells (VSMCs) and collagen levels were assessed based on the remaining samples (*n* = 4 per group) as follows: the samples were subjected to gradient dehydration with different concentrations of ethanol for 10 min, and then were treated with xylene to make tissues transparent, immersed in paraffin at 65 °C and embedded in paraffin wax. The sections of 4 μm were cut by a microtome (RM2016, Shanghai Leica Instrument Co., Ltd., Shanghai, China) and dried at 60 °C in an oven. The levels of α-SMA-positive VSMCs were assessed by immunostaining with primary antibody against α-SMA (1:200, Boster Biological Technology Co., Ltd., Pleasanton, USA), followed by HRP-labeled goat anti-mice-conjugated secondary antibody (1:200, SeraCare, Milford, CT, USA). Staining with Masson’s trichrome was used to delineate the collagen levels according to the instructions of the manufacturer (Wuhan Baiqiandu Biotechnology Co., Ltd., Wuhan, China). Finally, the slices were dehydrated, sealed with neutral gum, and viewed under a microscope. The percentage of the stained area (the stained area per total atherosclerotic lesion area) was calculated by Image-Pro Plus software.

### 2.6. Colon Histological Analysis

After being fixed in 4% paraformaldehyde (*v*/*v*), the colon tissue was dehydrated using a series of ethanol solutions, and then was treated with xylene to make the tissues transparent, immersed in paraffin at 65 °C, and embedded in paraffin wax. The sections of 4 μm were cut by a microtome and dried at 60 °C in an oven. Finally, the slices were dehydrated and dyed with hematoxylin and eosin (H&E), sealed with neutral gum, and viewed under a microscope.

### 2.7. Fecal Microbial Composition

Microbial DNA of fecal samples was extracted using a PowerSoil^®^ DNA Isolation kit (MO BIO, Carlsbad, CA, USA) according to the instructions of the manufacturer. The DNA concentration and purity were measured using Qubit 2.0 fluorometer (Life Technologies, Darmstadt, Germany), and the DNA extraction quality was evaluated using 1% agarose gel electrophoresis. The full-length 16S rRNA genes were amplified by PCR for SMRT sequencing. The 16S rRNA gene of bacteria was amplified using the forward 27F (5′-AGRGTTTGATYNTGGCTCAG-3′) and reverse 1492R (5′-TASGGHTACCTTGTTASGACTT-3′) primers. The PCR conditions were performed as follows: 1 cycle of 95 °C for 5 min, 30 cycles of 95 °C for 30 s, 50 °C for 30 s, and 72 °C for 1 min, and finally 1 cycle at 72 °C for 7 min. Multiple samples were mixed based on the amount of output data and fragment size required for each sample and purified with AMPure beads. According to the electrophoresis results and the concentrations detected by Qubit, as well as the number of mixed samples, fragment size, and total data output, the purified samples were mixed for a second time, and DNA damage repair and end repair were performed. Finally, the built library was sequenced using the PacBio SMRT II machine (PacBio, MenloPark, CA, USA). The original subreads were corrected to obtain Circular Consensus Sequencing (CCS) sequences (SMRT Link, version 8.0) (PacBio, MenloPark, CA, USA), and then using the Lima (version 1.7.0) software (PacBio, MenloPark, CA, USA), the CCS sequences of different samples were identified by barcode sequences, and chimeras were removed to obtain high-quality CCS sequences. Using USEARCH (version 10.0), the unique sequence set was classified into operational taxonomic units under the threshold of 97% identity. Principal coordinate analysis (PCoA) and nonmetric multidimensional scaling (NMDS) based on Bray-Curtis dissimilarity distances were used for analyzing structural changes of gut microbiota. The differences in the relative abundance of gut microbiota between the two groups were performed by the Wilcoxon rank-sum test with the metastats (http://metastats.cbcb.umd.edu/ (accessed on 3 February 2022)). Linear discriminant analysis (LDA) of effect size (LEfSe) was applied to determine the most discriminant taxa between groups (LDA score ≥ 3.0).

### 2.8. Non-Targeted Metabolomics Analysis

Cecal metabolites were detected using UHPLC-MS. Briefly, 50 mg of cecal content from each mouse (*n* = 5 per group) was weighed and the metabolites were extracted using 400 µL methanol solution (80%, *v*/*v*). The mixture settled at −20 °C and was treated by the high throughput tissue crusher Wonbio-96c (Shanghai Wanbo Biotechnology Co., Ltd., Shanghai, China) at 50 Hz for 6 min. The mixture was followed by vortex and ultrasound (40 kHz, 30 min, 5 °C) and was placed at −20 °C for 30 min. Then, the samples were centrifuged (13,000× *g*, 15 min, 4 °C) and the supernatant was analyzed by UHPLC-MS (Thermo, Waltham, MA, USA) equipped with an HSS T3 column (100 mm × 2.1 mm, 1.8 µm; Waters, Milford, CT, USA). Mobile phase A = 95% water + 5% acetonitrile (containing 0.1% formic acid), and B is 47.5% acetonitrile + 47.5% isopropanol + 5% water (containing 0.1% formic acid). The linear gradient was optimized as follows: 0% to 24.5% B (0 to 3.5 min), 24.5% to 65% B (3.5 to 5 min), 65% to 100% B (5 to 5.5 min), 100% B (5.5 to 7.4 min), 100% to 51.5% (7.4 to 7.6 min), 51.5 to 0% (7.6 to 7.8 min), and re-equilibration of the column with 0% B (7.8 to 10 min) at a flow rate of 0.4 mL/min and a column temperature of 40.0 °C. Mass spectrometry analysis was conducted in positive and negative ion modes and the mass scanning range is *m*/*z*: 70–1050. The source and ion transfer parameters applied were as follows: source heater = 400 °C, the capillary temperature = 320 °C, sheath gas = 40 arb, aux gas = 10 arb, ion spray voltage = 3.5 kV (positive ion mode) and 2.8 kV (negative ion mode). A quality control (QC) sample was injected every ten samples to monitor the reproducibility of the analytical platform. The raw data was imported into the metabolomics processing software Progenesis QI (Waters Corporation, Milford, CT, USA) for baseline filtering, peak identification, integration, retention time correction, peak alignment, and normalization, resulting in a data matrix with retention time, mass-to-charge ratio, and peak intensity. After removing isotope data, treating data with the 80% rule and treating data with the relative standard deviation (RSD) in the QC samples > 30%, the compounds were identified based on the exact mass number, secondary fragments and isotopic distribution, and were qualified using the Human Metabolome Database HMDB (http://www.hmdb.ca/ (accessed on 5 February 2022)) and METLIN database (https://metlin.scripps.edu/ (accessed on 5 February 2022)). The normalized data matrix of metabolomics was imported to the SIMCA Statistical Analysis software (version 13.0; Umetrics, Umea, Sweden) for unsupervised principal components analysis (PCA) to observe the overall sample distribution and orthogonal partial least-squares discriminant analysis (OPLS-DA) to identify metabolite differences between the groups. Metabolites with variable important in projection (VIP) scores > 1 and *p* < 0.05 were considered differential metabolites.

### 2.9. Statistical Analysis

All experimental data are presented as mean ± standard error of mean (SEM). Comparisons among groups were performed by one-way ANOVA followed by the Duncan test (SPSS v20.0, Chicago, IL, USA) and graphed by GraphPad Prism (San Diego, CA, USA). *p* < 0.05 was considered as statistically significant. Spearman correlation analysis was used to analyze the relationship between the biochemical indicators in serum and the relative abundance of gut microbiota.

## 3. Results

### 3.1. Effects of Capsaicin on HFD-Induced Atherosclerosis

To investigate the effect of capsaicin on the development of atherosclerosis, atherosclerotic lesions of the aortic sinus were evaluated. The result of Oil Red O-staining showed that the lesion area was decreased in the CAP group when compared with the HFD group (*p* < 0.01) (Figure 1B). The collagen levels in the aortic sinus were correlated with the stability of atherosclerotic plaques [17], and therefore we further evaluated the collagen levels by Masson’s trichrome staining. The results showed that the levels of collagen in the aortic sinus were increased in the CAP group when compared with the HFD group (*p* < 0.01) (Figure 1C), indicating that capsaicin administration was beneficial for the stability of atherosclerotic plaques. Furthermore, migration of VSMCs from the media into the intima contributed to the development of atherosclerosis [18], and immunofluorescence staining for the VSMCs marker α-SMA showed that the number of α-SMA-positive cells in the aortic sinus was decreased after treatment with capsaicin (*p* < 0.05) (Figure 1D). Importantly, the food intake of the mice in CAP group was not significantly different from that in the HFD group (Figure S1), indicating that these beneficial effects was not due to the energy intake differences. Interestingly, there were no significant differences in the above-mentioned indicators between the HFD group and the CAP+Abx group, indicating that depleting the gut microbiota by using antibiotic treatment abolished the anti-atherosclerosis effect of capsaicin.

### 3.2. Effects of Capsaicin on Serum Lipid Levels

**Figure 2:**
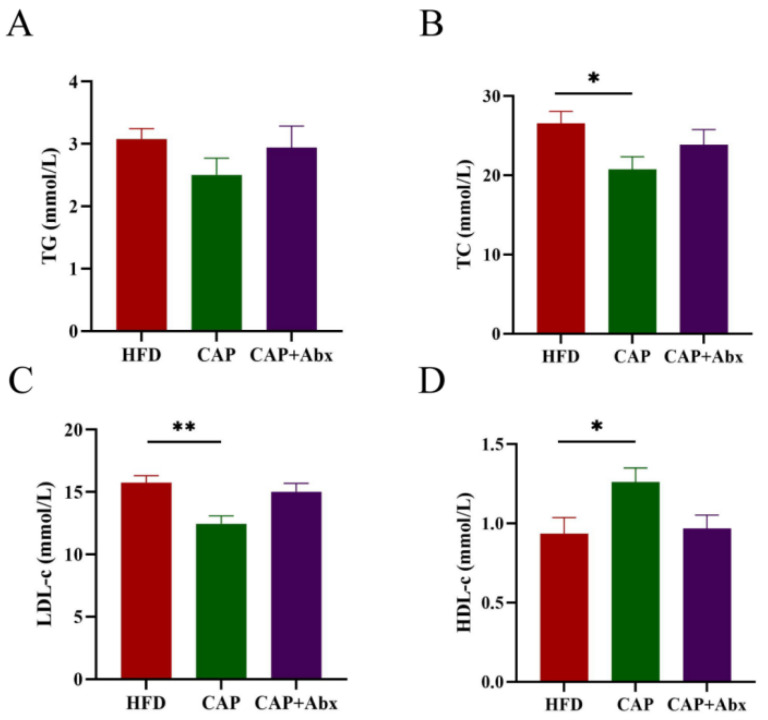
Effects of capsaicin administration and antibiotic intervention on serum lipid profiles. (**A**) TG; (**B**) TC; (**C**) LDL-c; (**D**) HDL-c. Data are expressed as mean ± SEM (*n* = 8 per group). * *p* < 0.05 and ** *p* < 0.01 compared with the HFD group. TC, total cholesterol; TG, total triglycerides; LDL-c, low-density lipoprotein-cholesterol; HDL-c, high-density lipoprotein-cholesterol.

The levels of blood lipids were strongly associated with the risk of cardiovascular disease [19], and thus we detected the serum TG, TC, LDL-c, and HDL-c levels in the three groups. Compared with the HFD group, the levels of TC but not TG were decreased in the CAP group (*p* < 0.05) (Figure 2A,B). Furthermore, our results showed that capsaicin administration reduced the levels of LDL-c (*p* < 0.01) and increased the levels of HDL-c (*p* < 0.05) in serum (Figure 2C,D). However, lipid levels in serum were not improved in the CAP+Abx group, suggesting that depleting the gut microbiota by using antibiotic treatment abolished the lipid-lowering effects of capsaicin.

### 3.3. Effects of Capsaicin on Inflammation

**Figure 3:**
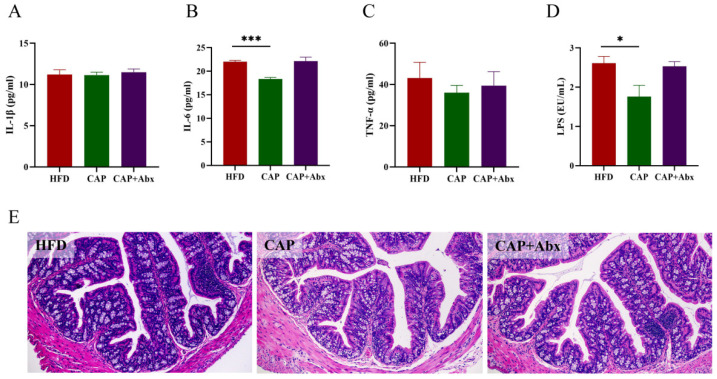
Effects of capsaicin administration and antibiotic intervention on serum proinflammatory cytokines levels and histopathology of the colon. (**A**) IL-1β; (**B**) IL-6; (**C**) TNF-α; (**D**) LPS; (**E**) H&E staining of colon tissue (100× magnification). Data are expressed as mean ± SEM (*n* = 8 per group). * *p* < 0.05 and *** *p* < 0.001 compared with the HFD group. IL-1β, interleukin-1β; IL-6, interleukin-6; TNF-α, tumor necrosis factor-α; LPS, lipopolysaccharide.

Considering the fact that inflammation has a key role in the development of atherosclerosis [20], we further investigated whether capsaicin administration could ameliorate inflammation in ApoE^−/−^ mice, and the levels of IL-1β, IL-6, TNF-α were detected in serum. The results showed that capsaicin administration could significantly decrease the levels of IL-6 compared with the HFD group (*p* < 0.0001). However, no significant differences in IL-1β and TNF-α levels were found between the groups (Figure 3A–C). LPS, a natural adjuvant synthesized by gram-negative bacteria in the intestine, plays a crucial role in the initiation of inflammation of several diseases including atherosclerosis. We observed that the levels of LPS were decreased in the CAP group, indicating that proinflammatory factor from the intestine was reduced after the treatment of capsaicin (*p* < 0.05) (Figure 3D). The levels of LPS in serum are closely related to the integrity of the intestinal mucosal barrier, and therefore we performed colon histological analysis. As shown in Figure 3E, the colon of the HFD group showed an inflammatory response, which was manifested as inflammatory cell infiltration and villi damage. However, after capsaicin administration, the inflammatory response in the colon was alleviate, indicating that capsaicin could improve the colon inflammation and protected the intestinal mucosal barrier in mice. As expected, the host inflammation and intestinal mucosal barrier were not improved in the CAP+Abx group, suggesting that depleting the gut microbiota by using antibiotic treatment abolished the anti-inflammatory effect and protective effects on the intestinal mucosal barrier.

### 3.4. Effects of Capsaicin on the Composition of Gut Microbiota

**Figure 4:**
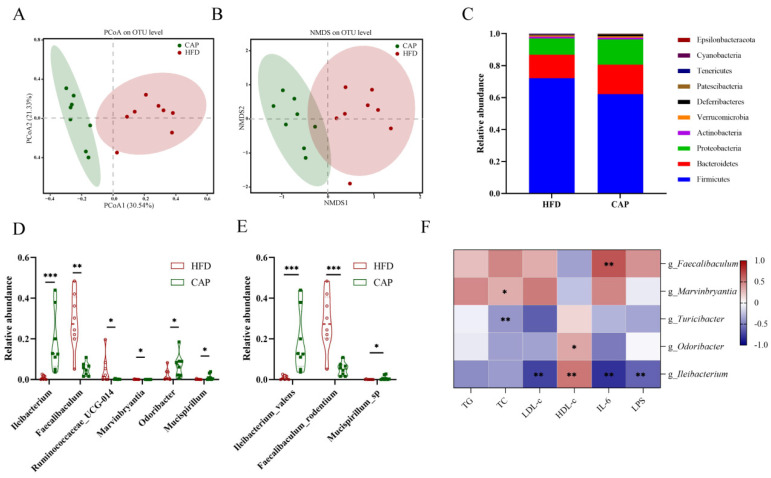
Effects of capsaicin administration on the composition of gut microbiota; (**A**) principal coordinate analysis (PcoA) score plot; (**B**) nonmetric multidimensional scaling (NMDS) score plot based on Bray–Curtis; (**C**) the relative abundance of gut microbiota at the phylum level; data are expressed as median and interquartile (*n* = 8 per group); (**D**) the relative abundance of gut microbiota at the genus level (*n* = 8 per group); (**E**) the relative abundance of gut microbiota at the species level (*n* = 8 per group); (**F**) the heatmap of Spearman’s correlation analysis between the gut microbiota and serum lipids and proinflammatory cytokines levels (*n* = 8 per group). * *p* < 0.05, ** *p* < 0.01 and *** *p* < 0.001 compared with the HFD group.

Based on the above results, we found that capsaicin administration could alleviate HFD-induced atherosclerosis and inflammation, while antibiotic intervention abolished these beneficial effects. Therefore, we speculated that gut microbiota may play a key role in the improvement of atherosclerosis by capsaicin, and the gut microbiota composition in the HFD group and the CAP group was measured by using SMRT sequencing technology. Actually, we tried to extract the bacterial DNA of gut microbiota in the CAP+Abx group, but no detectable bacterial DNA was found because of the antibiotic cocktail treatment. There was no difference in α-diversity (ACE, Chao1, Shannon, and Simpson indices) between the CAP group and the HFD group (Figure S2). PCoA and NMDS were used to evaluate the different composition of gut microbiota between the CAP group and the HFD group (Figure 4A,B). The results showed that the two groups formed separate clusters, indicating that capsaicin administration could significantly alter the composition of the gut microbiota. Furthermore, the relative abundance of gut microbiota was assessed at the phylum, genus, and species levels to identify the effect of capsaicin administration on gut microbiota. At the phylum level, *Firmicutes* and *Bacteroides* were two major phyla in the gut microbiota of ApoE^−/−^ mice. The abundance of phylum *Deferribacteres* was increased after capsaicin administration, but *Cyanobacteria* and *Tenericutes* showed the opposite trend (Figure 4C). At the genus level, the abundance of *Faecalibaculum* and *Marvinbryantia* was reduced in the CAP group, while the abundance of *Ileibacterium*, *Ruminococcaceae_UCG-014*, *Odoribacter*, and *Mucispirillum* was increased (Figure 4D). Depending on SMRT sequencing technology, the detection of gut microbiota can be accurate to the species level. It was found that the abundance of species *Faecalibaculum_rodentium* was reduced and the abundance of species *Ileibacterium_valens* and *Mucispirillum_sp* was increased in the CAP group (Figure 4E). Additionally, the LEfSe method was applied to the classification level of the species to phylum (Figure S3). LEfSe analysis showed that the HFD group was characterized by genera *Faecalibaculum* and *Marvinbryantia*, and their abundance was decreased in the CAP group. The CAP group was characterized by genera *Turicibacter*, *Odoribacter*, and *Ileibacterium*. We further analyzed the correlations of serum TG, TC, LDL-c, HDL-c, IL-6, and LPS levels with the abundance of gut microbiota by using Spearman correlation (Figure 4F). The results showed that the abundance of genera *Faecalibaculum* and *Marvinbryantia* was significantly positively correlated with serum IL-6 and LDL-c levels, respectively, while the genera *Odoribacter* and *Turicibacter* showed the opposite results. In addition, the relative abundance of genus *Ileibacterium* was not only significantly negatively correlated with serum LDL-c, IL-6, and LPS levels, but also significantly positively correlated with serum HDL-c levels.

### 3.5. Effects of Capsaicin on the Ccecal Metabolomic Profiles

**Figure 5:**
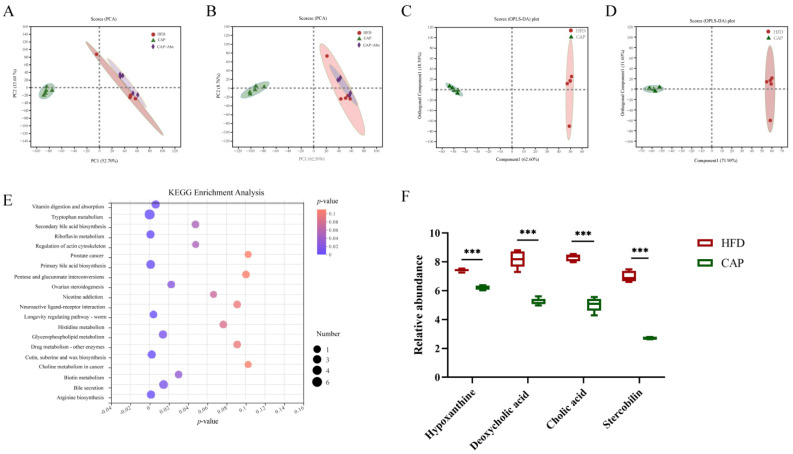
Effects of capsaicin administration on the cecal metabolomic profiles in mice. (**A**) Positive ion and (**B**) negative ion principal component analysis (PCA) score plots of cecal metabolomic profiles in the HFD, CAP, and CAP+Abx groups; (**C**) positive ion and (**D**) negative ion modes of orthogonal partial least squares discrimination analysis (OPLS-DA) score plots of cecal metabolomic profiles in the HFD and CAP groups (*n* = 5 per group); (**E**) KEGG enrichment analysis; (**F**) the abundance of cecal metabolites in the HFD and CAP groups; data are expressed as mean ± SEM (*n* = 5 per group). *** *p* < 0.001 compared with the HFD group.

To better explore the potential mechanism of gut microbiota in the improvement of atherosclerosis by capsaicin, we further investigated the cecal metabolites in ApoE^−/−^ mice using non-targeted metabolomics analysis. PCA under positive and negative ion mode electrospray ionization showed that the HFD group and the CAP group were clearly separated, indicating that the supplement of capsaicin significantly altered the metabolites. However, the CAP+Abx group showed a similar cluster to the HFD group, meaning a similar composition of metabolites between the two groups (Figure 5A,B). The OPLS-DA model was used to clearly distinguish each group in the paired comparisons (Figure 5C,D), and the model was verified (Figure S4A,B). VIP analysis (*p*-value < 0.05, VIP > 1) showed that a total of 438 differential metabolites were identified. These metabolites were mainly steroids and steroid derivatives, prenol lipids, organooxygen compounds, fatty acyls, and carboxylic acids and derivatives. Then, a KEGG pathway *p*-value analysis was performed and the results showed that the secondary bile acid biosynthesis was one of the metabolic pathways significantly affected by capsaicin (Figure 5E). Among all the differential metabolites observed, deoxycholic acid and cholic acid were associated with secondary bile acid biosynthesis. Additionally, the capsaicin administration also reduced the abundance of hypoxanthine and stercobilin (Figure 5F).

## 4. Discussion

**Figure 6:**
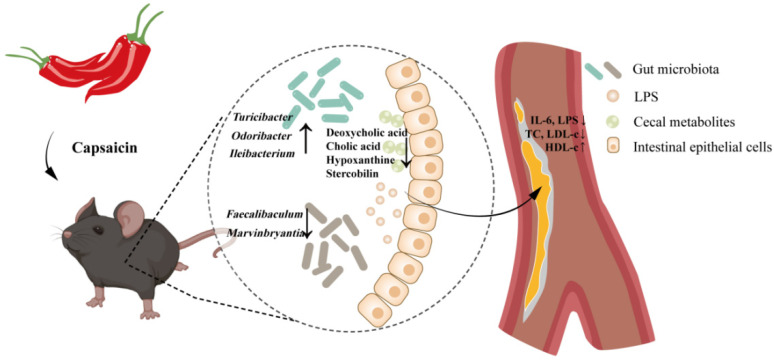
Capsaicin administration decreased the abundance of *Faecalibaculum* and *Marvinbryantia*, increased the abundance of *Turicibacter*, *Odoribacter*, and *Ileibacterium* in feces, and decreased the abundance of deoxycholic acid, cholic acid, hypoxanthine, and stercobilin in cecal content. These changes protected the integrity of the intestinal mucosal barrier, reduced the transportation of LPS from the gut into the blood, and reduced the levels of IL-6 and lipids in the serum, which further contributed to the improvement of atherosclerosis. However, all these beneficial effects of capsaicin were abolished after an antibiotic intervention. LPS, lipopolysaccharide; IL-6, interleukin-6.

Capsaicin is a natural alkaloid abundantly present in peppers with outstanding biological activities [8]. Animal experiments showed that capsaicin could alter the composition of gut microbiota, thereby losing weight, lowering serum lipids, and exerting an anti-inflammatory effect [14,21]. All of these effects can contribute to the prevention and adjuvant treatment of atherosclerosis, but whether the gut microbiota plays an important role in the anti-atherosclerosis effect of capsaicin has not been reported. In the present study, we evaluated the effects of capsaicin on ameliorating atherosclerosis in HFD-fed ApoE^−/−^ mice with intact and antibiotic-depleted gut microbiota, and we then explored the underlying mechanisms from the composition of gut microbiota and cecal metabolomic profiles (Figure 6). The results showed that capsaicin administration could significantly prevent the development of atherosclerosis and reduce serum lipid levels. Additionally, serum IL-6 and LPS levels were decreased, and HFD-induced inflammatory responses in intestinal mucosa were improved after capsaicin administration. However, all these beneficial effects of capsaicin were abolished after the antibiotic intervention. The results of gut microbiota showed that capsaicin significantly altered the composition of gut microbiota. LEfSe analysis showed that species *Ileibacterium_valens*, *Mucispirillum*_sp., and genera *Ruminococcaceae_UCG-014* and *Odoribacter* were increased after capsaicin treatment. Furthermore, the results of the analysis with cecal metabolites showed that capsaicin administration could significantly affect secondary bile acid biosynthesis and reduce the abundance of hypoxanthine, deoxycholic acid, cholic acid, and stercobilin.

The lesion of the aortic sinus is an important pathological feature of atherosclerosis [19]. We found that capsaicin supplementation significantly reduced the lesion area, prevented the immigration of α-SMA-positive VSMCs, and improved the stability of atherosclerotic plaques. These results indicated that capsaicin could significantly prevent the development of atherosclerosis, which is consistent with previous studies [9,11,12]. Atherosclerosis is usually accompanied by lipid metabolism disorders, especially by high levels of LDL-c which is the dominant form of atherogenic cholesterol [22]. The increased serum levels of TG, TC, and LDL-c were risk factors for atherosclerosis, while HDL-c was considered as “good cholesterol” to exert an atheroprotective effect [22]. Our study showed that capsaicin significantly reduced the levels of TC and LDL-c, and increased the levels of HDL-c in serum, which was supported by previous studies [23,24]. Furthermore, proinflammatory cytokines participate in the early phase of atherogenesis [25], and macrophages intake of excessive ox-LDL will form foam cells, which stimulate the secretion of more proinflammatory cytokines [26]; this vicious cycle further aggravates the development of atherosclerosis. Our study found that capsaicin could alleviate intestinal inflammation and protect the intestinal mucosal barrier. This effect reduced the transportation of LPS from the gut into the blood, further lowering serum IL-6 levels and thereby ameliorating atherosclerosis. However, antibiotic intervention prevented capsaicin from ameliorating atherosclerosis, improving serum lipid disorders and inflammation, suggesting that gut microbiota may be critical for capsaicin to ameliorate HFD-induced atherosclerosis.

Evidence from numerous studies showed that dysbiosis of gut microbiota could promote the development of atherosclerosis [27,28]. Previous studies suggested that capsaicin could modulate the composition of gut microbiota and against the gut microbiota dysbiosis induced by HFD [14,16], and thus we further investigated the composition of gut microbiota in ApoE^−/−^ mice after capsaicin. The results of LEfSe analysis showed that the genera *Faecalibaculum* and *Marvinbryantia* were the characteristic bacteria in the HFD group and significantly decreased in the CAP group. The abundance of genus *Faecalibaculum* was reported to be strongly positively correlated with serum lipids, proinflammatory cytokines, and intestinal permeability [29]. Another study pointed out that *Faecalibaculum* also correlated with the expression of lipid synthesis genes [30]. *Marvinbryantia* was considered a proinflammatory taxon and was associated with intestinal inflammation and bowel dysfunction [31,32], and was positively correlated with body weight [33]. Therefore, the decreased abundance of genera *Faecalibaculum* and *Marvinbryantia* in the CAP group may contribute to ameliorating the atherosclerosis. Furthermore, we found that several bacteria were significantly increased in the CAP group and had beneficial effects on atherosclerosis. In detail, *Turicibacter* has been reported to alleviate inflammatory responses and colitis [34,35], and was significantly increased after berberine intervention and ameliorated the development of atherosclerosis in an animal study [36]. *Odoribacter* was decreased in atherosclerotic patients and mice transplanted with proinflammatory microbiota [37,38]. *Ileibacterium* may contribute to the alleviation of HFD-induced hyperlipidemia [39], which is considered to be a critical factor contributing to atherosclerosis. Furthermore, *Turicibacter*, *Odoribacter*, and *Ileibacterium* were short chain fatty acid (SCFA)-producing bacteria. SCFA provided energy for intestinal epithelial cells and maintained intestinal homeostasis. Previous studies indicated that SCFA could reduce oxidative stress and improve the intestinal barrier [40,41]. Based on these results, we found that capsaicin reduced the abundance of proinflammatory bacteria and increased the abundance of anti-inflammatory bacteria. Therefore, we speculated that these changes in gut microbiota ameliorated the intestinal inflammation and protected the integrity of the intestinal mucosal barrier, which contributed to the lower serum LPS and IL-6 levels in the CAP group. Lower proinflammatory cytokines in the serum were further beneficial for ameliorating atherosclerosis. In addition, gut microbiota in the CAP group alleviated lipid metabolism disorders, thereby improving serum lipid levels including TC, LDL-c, and HDL-c, and further benefiting atherosclerosis. This point was confirmed by the results of Spearman’s correlation that the characteristic bacteria of the CAP group significantly negatively correlated with the serum lipids and proinflammatory cytokines levels.

To further investigate the potential mechanisms of capsaicin in ameliorating atherosclerosis, we performed a metabolomics analysis of cecal metabolites in ApoE^−/−^ mice. The results of PCA showed that the HFD group and the CAP group were clearly separated, and supplementation of capsaicin significantly reduced several metabolites, including deoxycholic acid, hypoxanthine, cholic acid, and stercobilin. Deoxycholic acid is a secondary bile acid produced by deconjugation and 7a-dehydroxylation by microbial enzymes in the colon [42]. A study showed that deoxycholic acid could promote the VSMCs proliferation and migration by activating c-jun N-terminal kinase, which supported our results that the levels of α-SMA-positive VSMCs were significantly decreased in the CAP group [43]. Additionally, higher intestinal deoxycholic acid concentrations could disrupt intestinal epithelial integrity due to the hydrophobic nature of deoxycholic acid [44]. Hypoxanthine was associated with intestinal inflammation and could induce atherosclerosis in ApoE^−/−^ mice [45,46]. Cholic acid may accelerate the development of atherosclerosis by activating oxidative stress-induced macrophage mobilization signals [47]. Stercobilin, a pigment in feces, was formed by intestinal bacteria, which could induce proinflammatory activities in mice [48]. These results indicated that capsaicin administration could significantly change the cecal metabolomic profile of ApoE^−/−^ mice and prevent the development of atherosclerosis. However, after the antibiotic intervention, the CAP+Abx group showed a similar cluster to the HFD group, implying that gut microbiota may contribute to these alterations after capsaicin administration.

There were some limitations in this research. First, the supplementation of capsaicin was only studied at a dose of 0.01% in the present study. Whether different doses of capsaicin could significantly ameliorate the development of atherosclerosis with the dose-effect relationship should be further studied. Second, in our study, we only investigated the effects of capsaicin on atherosclerosis in the version of gut microbiota and cecal metabolites, further potential mechanisms including atherosclerosis-related gene and protein expression need to be explored. Third, due to the lack of related research data and complex metabolic processes, the relationships between gut microbiota and cecal metabolites were not discussed in detail in this study.

## 5. Conclusions

In summary, our study found that dietary administration of capsaicin could prevent the development of atherosclerosis, reduce serum lipids, proinflammatory cytokines, and LPS levels, and improve HFD-induced inflammatory response in the colon. However, all these beneficial effects of capsaicin were abolished after an antibiotic intervention. Capsaicin administration also significantly altered the composition of gut microbiota and cecal metabolomic profiles, which might contribute to the improvement of atherosclerosis. Although the relationship of gut microbiota induced by capsaicin administration with atherosclerosis still needs further study, such as fecal bacteria transplantation and vitro fermentation experiments, the present study provides a new theoretical basis for the anti-atherosclerosis effect of capsaicin.

## Data Availability

Not applicable.

## Supplementary Materials

The following supporting information can be downloaded at: https://www.mdpi.com/article/10.3390/nu14204334/s1, Figure S1: Body weight changes in 18 weeks and food intake. Figure S2: Effects of capsaicin supplementation on alpha diversity of gut microbiota; Figure S3: Linear discriminant analysis (LDA ≥ 3.0) scores derived from LEfSe analysis (HFD vs CAP); Figure S4: Permutation test of serum metabolomic profiles in HFD and CAP groups under (A) positive ion and (B) negative ion modes.
