# Supplementary material for: Capsaicin Ameliorates High-Fat Diet-Induced Atherosclerosis in ApoE−/− Mice via Remodeling Gut Microbiota
Source: Nutrients. 2022 Oct 17;14(20):4334. doi: 10.3390/nu14204334 (PMC9611743; doi:10.3390/nu14204334)
Supplement: Supplementary file 1 [file nutrients-14-04334-s001.zip › nutrients-1964446-supplementary.pdf]

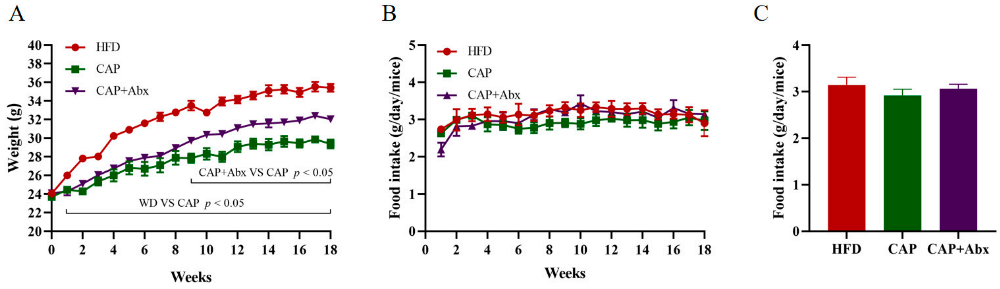

Figure S1. (**A**) Body weight changes in 18 weeks. (**B**) and (**C**) Food intake.

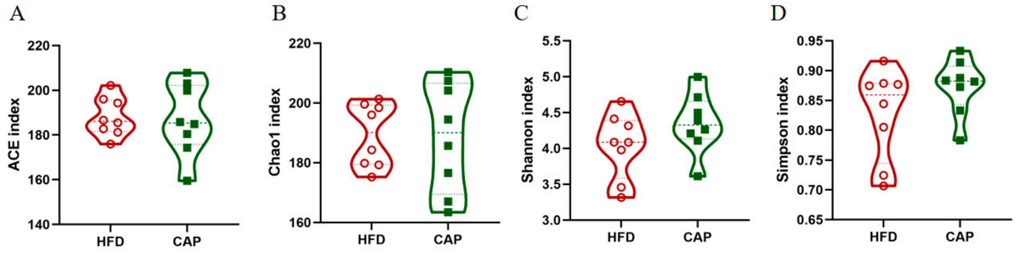

Figure S2 Effects of capsaicin supplementation on alpha diversity of gut microbiota (**A**) ACE index; (**B**) chao1 index; (**C**) shannon index; (**D**) simpson index

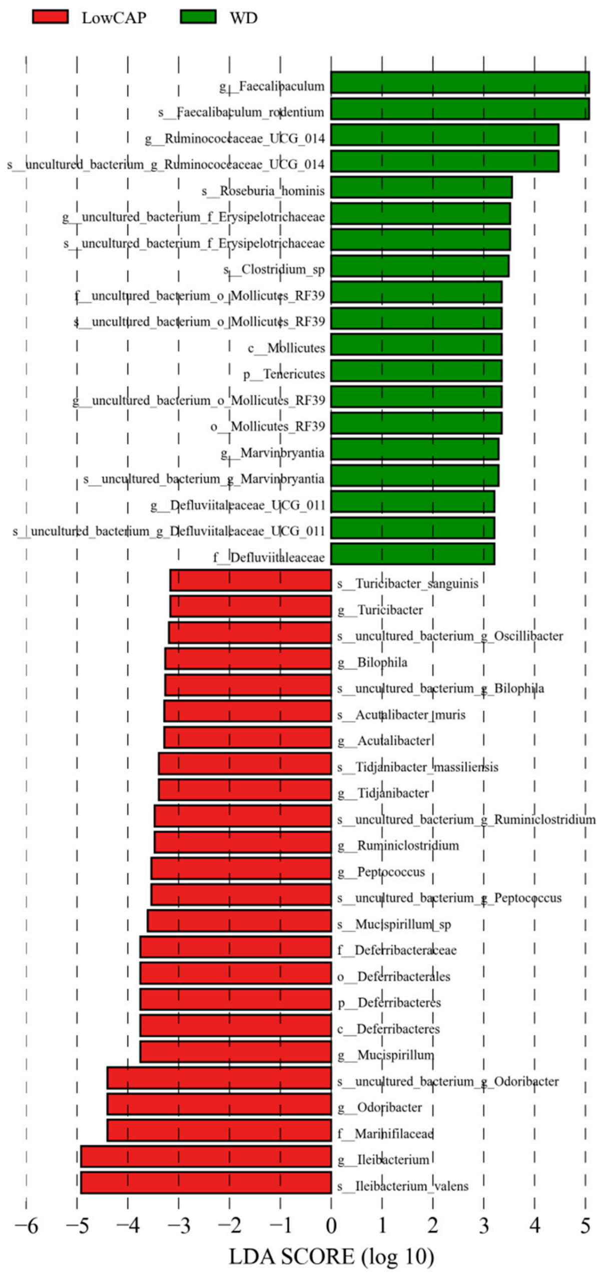

Figure S3 Linear discriminant analysis (LDA ≥ 3.0) scores derived from LEfSe analysis (HFD vs. CAP).

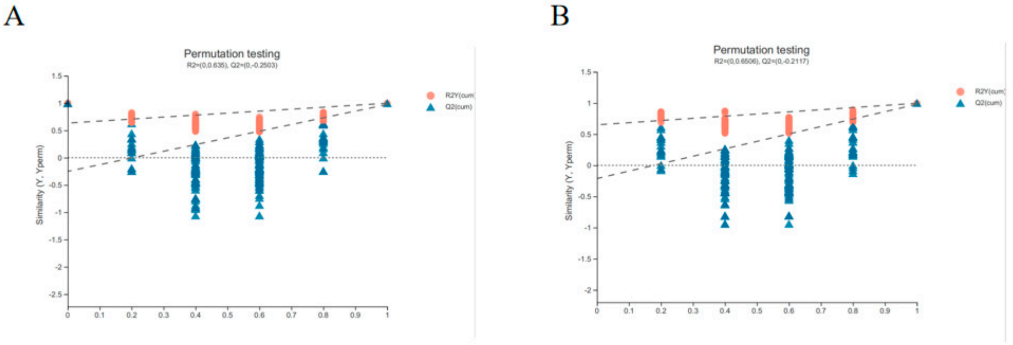

Figure S4 Permutation test of serum metabolomic profiles in HFD and CAP groups under (**A**) positive ion and (**B**) negative ion modes.
